# Can machine translation match human expertise? Quantifying the performance of large language models in the translation of patient-reported outcome measures (PROMs)

**DOI:** 10.1186/s41687-025-00926-w

**Published:** 2025-07-25

**Authors:** Sheng-Chieh Lu, Cai Xu, Manraj Kaur, Maria Orlando Edelen, Andrea Pusic, Chris Gibbons

**Affiliations:** 1 Department of Symptom Research, The University of Texas MD Anderson Cancer Center, 6565 MD Anderson Blvd, Houston, Texas USA; 2 Section of Patient-Centered Analytics, The University of Texas MD Anderson Cancer Center, Houston, Texas USA; 3 Department of Bioinformatics & Border Biomedical Research Center, The University of Texas at El Paso, El Paso, Texas USA; 4 Department of Surgery, Brigham and Women’s Hospital, Harvard Medical School, Boston, Massachusetts USA; 5 Department of Surgery, Patient Reported Outcomes Value and Experience (PROVE) Center Brigham and Women’s Hospital,, Boston, Massachusetts USA

**Keywords:** Patient-reported outcome measure, Large language models, Face-Q, Breast-Q, Machine translation

## Abstract

**Background:**

The rise in artificial intelligence tools, especially those competent at language interpretation and translation, enables opportunities to enhance patient-centered care. One might be the ability to rapidly and inexpensively create accurate translations of English language patient-reported outcome measures (PROMs) to facilitate global uptake. Currently, it is unclear if machine translation (MT) tools can produce sufficient translation quality for this purpose.

**Methodology:**

We used Generative Pretrained Transformer (GPT)-4, GPT-3.5, and Google Translate to translate the English versions of selected scales from the Breast-Q and Face-Q, two widely used PROMs assessing outcomes following breast and face reconstructive surgery, respectively. We used MT to forward and back translate the scales from English into Arabic, Vietnamese, Italian, Hungarian, Malay, and Dutch. We compared translation quality using the Metrics for Evaluation of Translation with Explicit Ordering (METEOR). We compared the scores between different translation versions using the Kruskal-Wallis test or analysis of variance as appropriate.

**Results:**

In forward translations, the METEOR scores significantly varied depending on target languages for all MT tools (*p* < 0.001), with GPT-4 having the highest scores in most languages. We detected significantly different scores among translators for all languages (*p* < .05), except for Italian (*p* = 0.59). In backward translations, MTs (GPT-4: 0.81 ± 0.10; GPT-3.5: 0.78 ± 0.12; Google Translate: 0.80 ± 0.06) received higher or compatible scores to human translations (0.76 ± 0.11) for all languages. The differences in backward translation scores by different forward translators were significant for all languages (*p* < 0.01; except for Italian, *p* = 0.2). The scores between different languages were also significantly different for all translators (*p* < 0.001).

**Conclusions:**

Our findings suggest that large language models provide high-quality PROM translations to support human translations to reduce costs. However, substituting human translation with MT is not advisable at the current stage.

## Introduction

Patient-Reported Outcome Measures (PROMs) have become essential tools for measuring health status, health-related quality of life, treatment efficacy, and satisfaction with the experience of care across various healthcare settings [1–4]. At the level of patients and clinicians (micro-level), PROMs are increasingly being used to set treatment expectations, understand patient experience of a condition, monitor patient health status longitudinally, and identify needs for more healthcare [5, 6]. At the level of hospital and other healthcare organizations (meso-level), PROMs are used for peer-benchmarking, to conduct comparative effectiveness research and quality improvement [7]. PROMs are also used to develop or improve healthcare policies on how healthcare is organized, delivered, and paid for at or across jurisdiction-level (macro-level) [8]. PROMs are also widely utilized to estimate outcomes of pharmaceutical or non-pharmaceutical interventions in large, international clinical trials [9]. Many of the PROMs that are used in the literature were created in English, and it is crucial to translate and culturally validate them to ensure their wide applicability and enable cross-cultural assessments and comparisons [10].

The importance of translating PROMs stems from several factors. First, translating PROMs allows for their use in diverse patient populations, enabling their use in research studies and clinical practice. This facilitates the participation of linguistically diverse individuals and promotes inclusivity in healthcare [11]. Second, it facilitates cross-cultural comparisons, providing valuable insights into the impact of treatments and interventions across different populations [12, 13]. It also allows for the accumulation of data that can be compared across different populations, thereby facilitating knowledge discovery and supporting evidence-based decision-making in healthcare. This process contributes to global standardization and increases the effectiveness of PROs reducing bias resulting from culturally irrelevant or misunderstood items [14]. Consequently, guidelines have been put in place to ensure a thorough translation process that accurately captures the desired cultural perspective [14, 15].

The rigorous translation and cross-cultural adaptation of PROM from English to the final target language involves six steps: forward translation, synthesis/reconciliation of translations, back translation, expert discussion, cognitive interview, and clinician consent [16–19]. Generating a high-quality translation of PROMs is a multistep procedure and can often be complex, labor-intensive, and time-consuming. Previous literature reviews [18, 20] on the methodology of PROM translations highlighted considerable variability in participant recruitment, teamwork, back-translation, consolidation, pretesting approaches, and the recommendation of international harmonization, reflecting different theoretical perspectives on equivalence, development approaches, and resource considerations. Further, the reliance on a single forward translation reduces validity and reliability. Adopting a multistep approach is strongly recommended to ensure quality [20]. The translation can be costly and is prone to inconsistencies or errors caused by human factors. Limited availability of qualified human translators with domain-specific knowledge and insufficient resources for large-scale translations further hinder the accessibility and efficiency of accurately translating PROMs. Altogether, these considerations highlight the need for alternative approaches that are advanced, cost-effective, and reliable to address potential errors and challenges.

Recent advances in large language models (LLMs) have revolutionized the field of machine translation [21]. Machine translation (MT) involves converting a given input in one language into an output that conveys the same meaning in a different language. This task encompasses both comprehending the sequence of the source input and generating an equivalent sequence in the target language [22]. A large language model is an advanced artificial intelligence system that leverages attention-based processes and understands human language by utilizing deep learning techniques and extensive training data. It generates coherent and contextually relevant text as a result. By leveraging advanced algorithms and vast amounts of training data, LLMs have demonstrated promising performance in various natural language processing tasks [21, 23]. The emergence of large language models (such as Generative Pre-trained Transformer (GPT) has opened new possibilities for efficient and effective machine translation of PROMs [24].

Machine translation may offer a number of advantages compared to human translation in terms of reduced processing time and costs. Furthermore, previous studies have found the translation accuracy and overall performance of machine translation based on artificial intelligence to be comparable to that of human translation [25, 26]. However, the specific application of LLMs in translating PROMs remains largely unexplored. It remains uncertain whether machine translation, powered by LLMs, can achieve a comparative translation quality equivalent to that of human expert translations for PROMs. Addressing this research gap is crucial to understand the potential and limitations of machine translation in the context of PROM instruments. Through this research, we aim to investigate the feasibility of LLMs in achieving comparable translation quality, which has implications for reducing translation costs, enhancing generalizability by shortening translation time, and improving the capacity to create accurate cross-cultural PRO assessments globally.

The objectives of this study are to investigate the potential of LLMs, including GPT-3.5 and GPT-4, to translate PROMs. We assessed the translation quality of machine translation using LLMs in comparison to human expert translations and the translations by Google Translate for these instruments. By harnessing MT, our goal is to reduce translation costs, improve the generalizability of PROM usage in different countries, and enhance the measurement of PROs. This paper contributes to the advancement of PROM translation methods, bridging the gap between machine translation and human expertise. Evaluating the translation quality of LLMs has implications for healthcare research, clinical practice, and cross-cultural healthcare evaluations.

## Materials and Methods

In this study, we forward translated the Satisfaction of Breasts – preoperative and postoperative scales of the BREAST-Q into Arabic, Vietnamese, and Italian, the Aging Appearance module of the FACE-Q into Hungarian and Malay, and the FACE-Q’s Satisfaction with Facial Appearance and Psychological functions into Dutch by GPT versions 3.5 and 4 and Google Translate. We then quantitatively compared the machine-translated versions to human-translated versions using Metrics for Evaluation of Translation with Explicit ORdering (METEOR) scoring to assess whether the machine translation is comparable to human translation. This study was deemed exempt from ethical consideration as no human subject was involved.

### Instruments and forward translation language selections

We selected the BREAST-Q [27] and FACE-Q [28] as example PROMs to understand the feasibility of machine translation in this study. Both instruments were developed and validated by a series of studies and are among the most widely used PROMs in plastic and reconstructive surgical care and research. The BREAST-Q Reconstruction module contains six scales that assess satisfaction and quality of life for women receiving breast surgery, and the FACE-Q Aesthetics module was designed to collect data from the perspectives of patients seeking minimally invasive or invasive facial aesthetics interventions. Both instruments are available in many languages, such as Spanish, Chinese, and German, and with many ongoing efforts to ensure that the PROMs are available in diverse languages.

Generative pre-trained transformer-4 was trained on 45 gigabytes of corpus from a variety of sources, including Wikipedia, news, books, and scientific journals published before or in 2021 [29]. Due to the uncertainty of what has been included in GPT − 4’s training data, we included only language versions of the instruments available after January 2022 to avoid potential bias resulted from the possibility that GPT-3.5 and GPT-4 had seen the human translation results.

Specifically, we included the Satisfaction with Breast – preoperative and postoperative scales of the BREAST-Q instruments, which contain 19 questions, and translated the questions into Arabic, Vietnamese, and Italian. For the FACE-Q, we translated its Aging Appearance section containing seven questions into Hungarian and Malay as well as the Satisfaction with Facial Appearance and Psychological Function scales, each comprising ten questions, into Dutch. The questions all have a similar structure, with the same section-specific prefix followed by the questions [27, 28]. The human-translated versions of these scales were obtained through the PROM developers (Q-Portfolio) and were used as the gold standard to evaluate the quality of machine translations. Although we were unable to compare MT quality across PROMs with the same language due to the uncertainty of GPT training data, the inclusion of a diverse group of languages allowed us to evaluate MT in languages representing different global regions with varying characters and writing directions.

## Translators

We employed three machine translators, including GPT-4, GPT-3.5, and Google Translate, to translate the selected scales from the BREAST-Q and FACE-Q to the selected languages. GPT-4 and − 3.5 are state-of-the-art large language models pre-trained with a huge corpus of internet text data using transformer architecture for unspecific tasks, such as text generation, translation, and summarization. The primary function of the models is to generate human-like responses that best answer the given inputs. Although the models can be future fine-turned on a specific task, such as translation, with a much smaller dataset to improve their performance, it has been shown that GPT achieved better scores in a wide variety of exams, such as Scholastic aptitude test (SAT) Evidence-based reading and writing and Graduate Record Examination than most human test takers and outperformed most state-of-the-art language processing system in tradition NLP benchmarks [29]. Therefore, we used GPT-4 and − 3.5 without further fine-tuning them for instrument translation.

Generative pre-trained transformer-4 is the next generation of GPT-3.5 with improved capacities in language understanding and generation because of the use of a larger training dataset and an increase of parameters [29]. However, as of August 2023, an official release of GPT-4 was not yet available, and there was no solid knowledge concerning the performance comparison between GPT-4 and − 3.5. Thus, we tested both models in this study.

Another machine translator we tested is Google Translate. Google Translate is a free translation service provided by Google leveraging deep learning techniques. Researchers have argued the potential of Google Translate for healthcare-related survey instrument translation and tested the usability of the tool in facilitating medical communication [30]. Nevertheless, we found no reports concerning the accuracy and usefulness of Google Translate in PROM translation and the comparison between Google Translate and GPT models for the task.

### Study processes

We conduct the translation following the Functional Assessment of Chronic Illness Therapy translation methodology to translate the instruments into target languages and evaluate the translation quality [31, 32]. We did not proceed to the testing of translations and further steps, as the primary purpose was to pilot the potential of machine translation and compare the translations using different approaches. We first forward-translated the instruments to target languages using GPT-3.5, GPT-4, and Google Translate. Although GPT can be used without configuration, there were several configurable parameters to control model behavior. We demonstrated the function of each parameter and our configuration when performing translations using GPT in Table 1. There was a prefix staging up the context for each section to allow a better understanding of each question in the section. In this analysis, we translated the instruments using a sentence-by-sentence approach and considered the prefixes as part of the questions. Thus, the actual questions that were fed to a machine translator included their prefixes plus the questions. For example, we asked GPT to translate “With your breast area in mind, in the past week, how satisfied or dissatisfied have you been with: How you look in the mirror clothed?” rather than “How you look in the mirror clothed?” only.

**Table 1:** Generative pre-trained transformer-4 and − 3.5 parameters and configurations

Parameters	Function	GPT-4 configuration	GPT-3.5 configuration
Model	Indicates model to use	gpt-4	gpt-3.5-turbo
Messages*	Messages for the machine	{“role”: “user”, “content”: f”Please translate this question into {Target_language}: {Original PROM item}”}	{“role”: “user”, “content”: f”Please translate this question into {Target_language}: {Original PROM item}”}
Temperature	Indicates sampling temperature to use. A higher temperature value makes the model output more random	0	0
Max_tokens	Indicates the maximum length of responses generated by the model	1000	1000
Frequency_penalty	Indicates the penalty penalizing frequent tokens in the text	0	0

*Messages varied depending on target languages and PROM items to be translated

Abbreviations: GPT: generative pre-trained transformer; PROM: patient-reported outcome measure

We then compared each translation version to the corresponding human translation using Metrics for Evaluation of Translation with Explicit ORdering (METEOR score). The metric enables automated, objective scores for scientific evaluations of machine translation quality [33, 34]. For instance, researchers used METEOR to compare the semantic consistency between the translations of medical device adverse event terminology using a variety of deep learning models and by human experts. Their result showed the alignment between METEOR and human grader results [35]. The metric assesses translation quality by considering several aspects including direct word, stem, synonym, and paraphrase matching and alignment features (such as chunkiness) between the translations and references [36]. The METEOR score can be any value between 0 and 1, with 1 indicating a perfect match. We used the default parameter settings defined by the package used for the METEOR score calculation.

In addition to forward translation, we performed backward translation on each translation version (Human translation, GPT-4, GPT-3.5, and Google Translate) back to English using GPT-4, GPT-3.5, and Google Translate as the backward translators. We then use the METEOR score to understand the comparison between each backward-translated version and its original English version.

### Data analysis

We conducted all forward and backward translations using the OpenAI package (version 0.27.8) for GPT translation and the deep_translator package (version 1.9.1) for Google Translate service in Python 3.10 [37]. For METEOR score calculation, we employed the NLTK python package (version 3.8.1). For the analysis of the METEOR evaluation results, we compared the scores using both visual inspections and statistical tests to determine whether the machine translations were statistically comparable to the human translations. For statistical analysis, we used simple descriptive analysis to describe our data and analysis of variance (ANOVA) to compare the differences in METEOR scores between translators and languages. The Kruskal-Wallis test was used for the comparisons when the assumptions of ANOVA were not met. We conducted post hoc pairwise comparisons using the Tukey adjustment when the ANOVA showed significance and the Dunn test with Holm adjustment when the Kruskal-Wallis test showed significance. We conducted all descriptive and statistical analyses using the R statistical software package [38].

## Results

### Forward translation

**Fig. 1:**
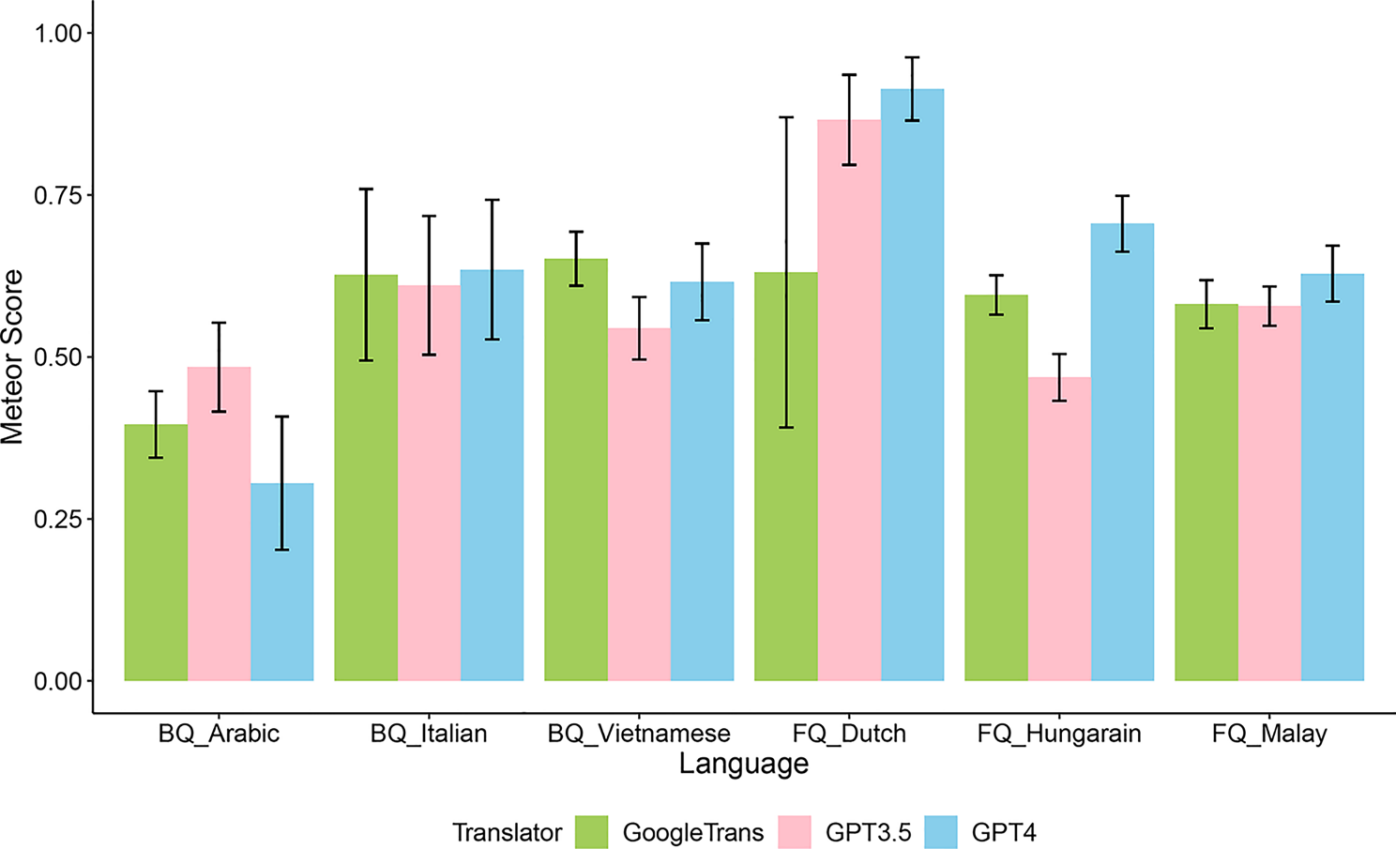
METEOR scores for forward translations by translators for each language. BQ: BREAST-Q; FQ: FACE-Q; GPT: generative pre-trained transformer; METEOR: Metrics for Evaluation of Translation with Explicit ORdering

We forward translated a total of 19 questions from the BREAST-Q into Arabic, Vietnamese, and Italian, 7 questions from the FACE-Q into Hungarian and Malay, and 20 questions from FACE-Q into Dutch using GPT-4, GPT-3.5, and Google translate. We present the METEOR scores by target language and translator in Fig. 1. In summary, GPT-4 had the highest scores in all languages except for Arabic and Vietnamese, and all translators received a mean METEOR score < 0.5 in Arabic translation.

Considering the performance in different languages for each translator, the METEOR scores significantly varied depending on the target languages for all translators (all p < .001), with post hoc pairwise comparisons showing that GPT-4 and − 3.5 had the best scores on Dutch, and Google Translate had a significantly lower score on Arabic than other languages (Table 2).

**Table 2:** Comparison pf METEOR score for forward translations by language within each translator

Language	Arabic		Vietnamese		Italian		Hungarian		Malay		Dutch		Statistics
Translator	Mean	SD	Mean	SD	Mean	SD	Mean	SD	Mean	SD	Mean	SD	
GPT – 4	0.31^a^	0.10	0.62^b^	0.06	0.64^b,c^	0.11	0.71^c^	0.04	0.63^b^	0.04	0.91^d^	0.05	*X*^2^ = 73.42, df = 5, *p* < 0.001
GPT – 3.5	0.48^a^	0.07	0.54^b^	0.05	0.61^c^	0.11	0.47^a^	0.04	0.58^b,c^	0.03	0.87^d^	0.07	*X*^2^ = 65.72, df = 5, *p* < 0.001
Google Translate	0.40 ^a^	0.05	0.65^b^	0.04	0.63^b^	0.13	0.60^b^	0.03	0.58^b^	0.04	0.63^b^	0.24	*X*^2^ = 39.47, df = 5, *p* < 0.001

^*a,b,c,d*^: for each translator, languages not sharing any letter are significantly different by the post hoc tests with adjustment.

Abbreviations: GPT: generative pre-trained transformer; METEOR: Metrics for Evaluation of Translation with Explicit ORdering.

For each language, we found significant differences in METEOR scores between translators for all languages (p = 0.04 for Malay and p < 0.001 for all others) as well, except for Italian (p = 0.59). Post hoc pairwise comparisons indicated that GPT-4 had a better score than others when the target languages were Dutch (0.91 ± 0.05) and Hungarian (0.71 ± 0.04), GPT-3.5 had a better score in Arabic (0.48 ± 0.07), and Google Translate had a better score for Vietnamese (0.65 ± 0.04). We detected no significant difference in pairwise comparisons for Malay (Table 3).

**Table 3:** Comparison of METEOR score for forward translation between translators for each language

Translators	GPT-4		GPT-3.5		Google Translate		Statistics
Languages	Mean	SD	Mean	SD	Mean	SD	
BREAST-Q							
Arabic (n = 19)	0.31^a^	0.10	0.48^c^	0.07	0.40^b^	0.05	*X*^2^ = 26.5, df = 2, *p* < 0.001
Vietnamese (n = 19)	0.62^b^	0.06	0.54^a^	0.05	0.65^c^	0.04	*X*^2^ = 24.1, df = 2, *p* < 0.001
Italian (n = 19)	0.64	0.11	0.61	0.11	0.63	0.13	*X*^2^ = 1.07, df = 2, *p* = 0.59
FACE-Q							
Hungarian (n = 7)	0.71^c^	0.04	0.47^a^	0.04	0.60^b^	0.03	F(2, 18) = 72.1, *p* < 0.001
Malay (n = 7)	0.63^a^	0.04	0.58^a^	0.03	0.58^a^	0.04	F(2, 18) = 4.0, *p* = 0.04
Dutch (n = 20)	0.91^b^	0.05	0.87^b^	0.07	0.63^a^	0.24	*X*^2^ = 17.1, df = 2, *p* < 0.001

^*a,b,c*^: for each language, translators not sharing any letter are significantly different by the post hoc tests with adjustment.

Abbreviations: GPT: generative pre-trained transformer; METEOR: Metrics for Evaluation of Translation with Explicit ORdering.

### Backward translation

**Fig. 2:**
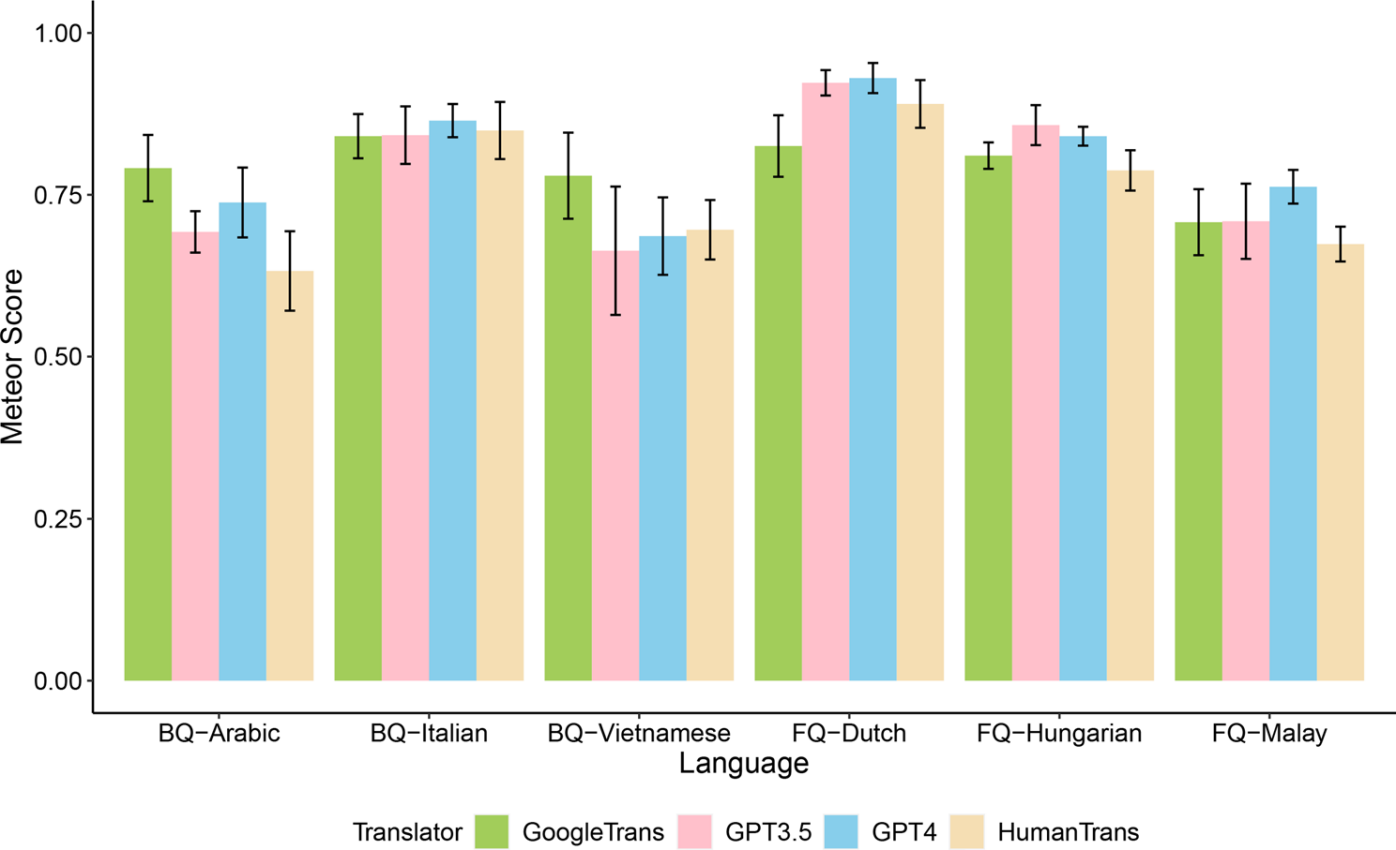
Mean METEOR scores for backward translations. BQ: BREAST-Q; FQ: FACE-Q; GPT: generative pre-trained transformer; METEOR: Metrics for Evaluation of Translation with Explicit ORdering

For the performance analysis of backward translation, we averaged the METEOR scores across backward translators for each language. Figure 2 shows the backward translation METEOR scores by translators and languages. All scores for backward translations were much higher than the scores for forward translations, regardless of languages and translators. Machine translations (GPT-4: 0.81 ± 0.10; GPT-3.5: 0.78 ± 0.12; Google Translate: 0.80 ± 0.06) received higher or compatible scores to human translations (0.76 ± 0.11) in the backward translation evaluation for all languages.

Further comparing the METEOR scores for backward translations for each language by forward translator, we found that different forward translators received significantly different scores in the backward translation assessment for all other languages (p < 0.01) except for Italian (p = 0.20) (Table 4). Pairwise comparisons suggested that GPT-4 had the best translation quality in terms of METEOR score in Dutch (0.93 ± 0.02) and Malay (0.76 ± 0.03), Google Translate performs well in Arabic (0.79 ± 0.05) and Vietnamese (0.78 ± 0.07). Human translation received the lowest scores in the analysis for most languages except for Italian and Dutch.

**Table 4:** Comparison of mean backward translations METEOR scores by translator for each language

Translators	Human		GPT-4		GPT-3.5		Google Translate		Statistics
Languages	Mean	SD	Mean	SD	Mean	SD	Mean	SD	
BREAST-Q									
Arabic (n = 19)	0.63^a^	0.06	0.74^c^	0.05	0.69^b^	0.03	0.79^d^	0.05	F(3, 72) = 33.56, *p* < 0.001
Vietnamese (n = 19)	0.70^a^	0.05	0.69^a^	0.06	0.66^a^	0.10	0.78 ^b^	0.07	*X*^2^ = 22.74, df = 3, *p* < 0.001
Italian (n = 19)	0.85	0.04	0.87	0.03	0.84	0.04	0.84	0.03	*X*^2^ = 4.64, df = 3, *p* = 0.20
FACE-Q									
Hungarian (n = 7)	0.79^a^	0.03	0.84^b,c^	0.01	0.86^c^	0.03	0.81^a,b^	0.02	F(3, 24) = 10.63, *p* < 0.001
Malay (n = 7)	0.67^a^	0.03	0.76^b^	0.03	0.71^a,b^	0.06	0.71^a,b^	0.05	*X*^2^ = 12.90, df = 2, *p* = 0.005
Dutch (n = 20)	0.89^b^	0.04	0.93^c^	0.02	0.92^c^	0.02	0.83^a^	0.05	*X*^2^ = 46.63, df = 3, *p* < 0.001

^*a,b,c,d*^: for each language, translators not sharing any letter are significantly different by the post hoc tests with adjustment

Abbreviations: GPT: generative pre-trained transformer; METEOR: Metrics for Evaluation of Translation with Explicit ORdering.

Backward translation assessment also suggested that the translation quality of each translator varied depending on the target language (all p < 0.001; Table 5). All translators demonstrated higher translation quality in Dutch compared to their translations for other languages (Human: 0.89 ± 0.04; GPT-4: 0.93 ± 0.02; GPT-3.5: 0.92 ± 0.01; Google Translate: 0.83 ± 0.05). However, the languages with the lowest scores in the analysis differed by the translators (Table 5).

**Table 5:** Comparison of mean backward translations METEOR scores by language for each translator

Language	Arabic		Vietnamese		Italian		Hungarian		Malay		Dutch		Statistics
Translator	Mean	SD	Mean	SD	Mean	SD	Mean	SD	Mean	SD	Mean	SD	
Human	0.63^a^	0.06	0.70^b^	0.05	0.85^d^	0.04	0.79^c^	0.03	0.67^a,b^	0.03	0.89^d^	0.04	F(5, 85) = 89.98, *p* < 0.001
GPT – 4	0.74^b^	0.05	0.69^a^	0.06	0.87^c^	0.03	0.84^c^	0.01	0.76^b^	0.03	0.93^d^	0.02	*X*^2^ = 78.44, df = 5, *p* < 0.001
GPT – 3.5	0.69^a^	0.03	0.66^a^	0.10	0.84^b^	0.04	0.86^b^	0.03	0.71^a^	0.06	0.92^c^	0.01	*X*^2^ = 70.85, df = 5, *p* < 0.001
Google Translate	0.78^b^	0.05	0.78^a,b^	0.07	0.84^c^	0.03	0.81^b,c^	0.02	0.71^a^	0.05	0.83^b,c^	0.05	*X*^2^ = 27.42, df = 5, *p* < 0.001

^*a,b,c,d*^: for each translator, languages not sharing any letter are significantly different by the post hoc tests with adjustment.

Abbreviations: GPT: generative pre-trained transformer; METEOR: Metrics for Evaluation of Translation with Explicit ORdering

## Discussion

We found that GPT-4 achieved a higher mean METEOR score across the languages tested in the backward translation assessment, aligning with many reports suggesting that AI-based machine translators can provide translations for various types of corpus and languages with comparable quality to human professionals [39]. This finding does not eliminate the need for human professionals to translate assessment tools that may be used to inform high-stakes decisions, such as treatment planning at the current stage. In fact, the lower METEOR scores for human translations could result from the cultural adaptation processes for cultural appropriateness enhancement. Alternatively, our findings support the notion that a combination of human and machine translations may result in better-quality PROM translations with reduced resource needs [40]. Future research is needed for the evidence on whether machine translators can provide comparable translation quality to human translators in terms of fluency, semantic clarity and equivalence, cultural appropriateness, and ethical considerations to allow a replacement. In addition, there is a need for a discussion enabling guidance on best practices for using AI in PROM translation purposes.

Previous research comparing GPTs to commercial machine translators, including Google Translate, on several translation tasks revealed that GPTs outperformed commercial machine translators in high-resource European languages but not in low-resource languages and concluded that GPT can be a good translator [26]. Our findings added confidence to the evidence by examining different sets of languages and translation tasks. The lag behind mature commercial machine translators for GPTs in low-resourced language translations could be due to the use of web text as training datasets for GPT development. As the GPTs we examined in this study were default models trained for unspecific purposes, further fine-tuning the models on low-resource languages for PROM translation tasks should significantly improve their translation quality [29].

Although GPTs achieved good METEOR scores across languages and instruments, we found that the scores failed to catch nuances in wording and sentence structure that may entirely change the semantics of a sentence when comparing the backward translations of GPT-4 translations to the original English instruments. For example, in FACE-Q, the backward translation of “I feel *okay* about myself” from Dutch by GPT-4 was “I feel *good* about myself.” As expertise in the included target languages was not available to the team, we only made observations on comparing backward translations to the original English instruments. It was not possible for us to confirm whether the nuances also existed in the forwarding translations by machines as compared to human translations. Nevertheless, this finding aligned with previous reports suggesting that the use of automated translation quality graders alone may not be sufficient to evaluate the quality of machine translations [33, 41]. The finding may also highlight a concern in the established PROM translation methodology in which English backward translations are evaluated for forward translation appropriateness. Whether the backward translation truly represents the forward translation is difficult to evaluate without the assistance of bilingual human experts. This concern represents another potential application of LLMs to ensure the reconciliation between forward and backward translations.

Our finding that machine translations could deviate from the original items of an instrument due to the small nuances in wording and sentence structure has implications for future PROM development. The use of machine translations has become prevalent nowadays because of the rapid development of language models capable of providing quality translation and their availability. Although it is not advisable to use machine-translated PROMs to collect patient data, it is inevitable that patients and researchers may require machine translations to aid with their understanding of instruments not in their languages [40]. In such cases, inaccurate translation by machines may result in suboptimal use of the instruments. Therefore, researchers should make extra considerations when developing instruments to avoid the opportunity for machines to generate semantically inequivalent translations. Collaboration of machine translators in instrument development to avoid the use of wording that may result in mistranslation may avoid the misuse of the instrument and allow machines to generate better translations to reduce translation resources needed. There is also potential to use LLMs to translate the feedback from native speakers of the target language in the cognitive interview step to enhance the understanding of their feedback for translation optimization.

The METEOR scores of our forward translations were notably lower than backward translations for most of the languages examined. The result could be attributed to the partial support of the METEOR scores for evaluating the similarity between sentences in most languages other than English [42]. METEOR evaluated translation quality by considering exact word, stem, and synonym matches between machine translations and human translations [36]. As the current implementation of METEOR did not support stem and synonym matches for all languages [42], all METEOR scores for our forward translations were solely based on the extract word match, which may not accurately reflect the translation quality. Therefore, the low METEOR scores for our forward translation do not indicate the low translation quality of GPTs. Alternatively, we considered that the good scores for backward translations by GPTs indicated the good quality of forward machine translations. Although researchers have made substantial efforts to establish lexical databases of words, senses, and semantic relations between words for non-English languages [43], further effort is needed to enable easy implementation of machine translation quality assessment based on these databases to allow better translation quality evaluation.

This study has limitations. First, due to the uncertainty in GPT’s training data, we were unable to examine a wide variety of instruments with more commonly used language versions published before 2021 and compared translation quality on the same instruments across languages. Also, despite the observed optimal scores in backward translations for GPT translation, we were unable to determine whether the translation quality is sufficient because of the lack of expertise in the examined target languages for a thorough comparison between machine translations and human translations. Lastly, reproducing our analyses reported could be challenging as GPTs could generate different translations for different attempts to translate the same sentence, despite the use of the same configuration. Also, varying the GPT translation prompts may alter the translation results. In this study, we did not examine the influences of different GPT setups nor determine the optimal GPT prompt. Additionally, we examined only a few state-of-the-art language models in this study at the time the experiment was conducted. As the area rapidly grows, many advanced LLM solutions specifically designed for translation tasks become available and have the potential to provide higher translation quality at an additional cost, which may limit their adoption by some research groups and institutions. Nevertheless, this study provides preliminary data assessing the usefulness of LLMs in PROM translations and evidence supporting further evaluations of PROM translations using other advanced LLM tools by human graders. To expand on this work, we are conducting a survey with bilingual cancer patients to evaluate the quality of LLMs in PROM translations using validated translation quality assessment metrics [44]. Incorporating human evaluations will enable a comprehensive understanding of LLM suitability in PROM translations.

## Conclusions

Translations of PROMs into various languages are essential to enable cross-cultural comparable data from patient perspectives but are resource intensive. We quantitatively characterized the translation quality of recently developed GPTs for the translation of two PROM instruments into six languages. Our findings suggest that LLMs can provide high-quality translations for PROMs to support human translations in terms of vocabulary equivalence. However, substituting human translation with machine translation is not advisable, considering the current state of LLMs. Further studies are needed to (1) qualitatively compare machine translations by LLMs to human translations to affirm LLMs’ usability in PROM translations by human graders; (2) fine-tune LLMs with text in low-resource languages to enhance their translation quality; and (3) enhance automatic machine translation evaluation algorithm to support objective quality appraisal of non-English translations.

## Data Availability

All data generated or analyzed during this study are included in this published article and its supplementary information files.

## Abbreviations

ANOVA: Analysis of variance
GPT: Generative pre-trained transformer
LLM: Large language model
METEOR: Metrics for evaluation of translation with explicit ordering
MT: Machine translation
NLP: Natural language processing
PROM: Patient-reported outcome measure
SAT: Scholastic Aptitude Test
